# Clinical Outcomes of 1.5-Stage Arthroplasty for Native Joint Septic Arthritis of the Hip and Knee: A Retrospective Cohort Study

**DOI:** 10.1016/j.artd.2026.102038

**Published:** 2026-05-20

**Authors:** Matthias Wittauer, Conor Farrell, Nikhil Sabharwal, Laurens Manning, Benjamin Clark, Piers J. Yates, Christopher W. Jones

**Affiliations:** a Department of Orthopaedic Surgery, Fremantle and Fiona Stanley Hospitals, Perth, Australia; b The Orthopaedic Research Foundation of Western Australia (ORFWA), Perth, Australia; c University of Basel, Basel, Switzerland; d Tallaght University Hospital, Dublin, Ireland; e Department of Infectious Diseases, Fiona Stanley Hospital, Perth, Australia; f School of Medicine, University of Western Australia, Perth, Australia

**Keywords:** Septic arthritis, Osteoarthritis, Infection, Arthroplasty, Hip, Knee

## Abstract

**Background:**

Native joint septic arthritis (NJSA) requires joint excision and arthroplasty when debridement fails or irreversible joint damage is present. Traditionally, this is performed as a staged procedure with a spacer and antibiotics prior to definitive arthroplasty. While effective, this approach is associated with multiple operations, prolonged treatment, and increased morbidity. The 1.5-stage arthroplasty has recently emerged as a potential alternative in treating periprosthetic joint infections, but its role in NJSA remains unexplored. This study evaluated the clinical outcomes of 1.5-stage arthroplasties in hips and knees for NJSA.

**Methods:**

A retrospective analysis was conducted on patients who underwent a 1.5-stage (n = 45) arthroplasty for NJSA between 2015 and 2024. Primary outcome was the revision rate at 12 months postoperatively. Secondary outcomes included surgical complication rates, length of hospital stay, rate of progression to second stage, 30-day emergency department and hospital readmission rates, and 1-year mortality.

**Results:**

At 1 year, the revision rate was 26.7%, with only 2 revisions performed for surgical complications (6.7%). Overall, 24.4% of patients progressed to a planned second stage within 1 year, primarily for functional optimization. The median length of stay was 13.5 days (interquartile range: 8–16). Thirty-day emergency department presentations (11.1%), readmissions (6.7%), and 1-year mortality (4.4%) were low and did not differ between hip and knee cohorts. No baseline demographic or clinical factors were significantly associated with revision risk.

**Conclusions:**

These findings suggest that 1.5-stage arthroplasty is a safe and effective option for NJSA, potentially avoiding additional surgery in selected patients.

## Introduction

Native joint septic arthritis (NJSA) is an uncommon but serious condition associated with significant morbidity and mortality [1]. If not promptly and effectively treated, NJSA can lead to rapid and irreversible destruction of articular cartilage, resulting in joint contractures, chronic pain, and permanent loss of function in up to 40% of cases [2]. Mortality rates at 2 years are estimated at around 11% for monoarticular infections, rising to 50% in polyarticular cases [3].

Standard management includes joint aspiration, targeted antibiotic therapy, and surgical debridement [4]. However, infection control often fails after a single debridement, particularly in older patients or those with degenerative joint disease [5]. In cases of persistent infection with joint destruction, total joint arthroplasty may become necessary. However, these patients constitute a high-risk group for periprosthetic joint infection (PJI), with reported rates ranging from 8% to 12% [[6], [7], [8], [9]], particularly when arthroplasty is performed within 1 year of the initial NJSA episode [10,11].

Recent interest has grown in 1.5-stage procedures using articulating spacers for the treatment of NJSA [12]. This technique was developed as a modification of the traditional 2-stage approach for managing chronic PJI, aiming to balance effective infection control with improved patient function with a single operation [13,14]. In the context of NJSA, the 2-stage procedure involves placement of a temporary cement spacer, together with a period of antibiotic therapy, followed by delayed reimplantation of a definitive prosthesis. By contrast, the 1.5-stage approach utilizes an antibiotic-laden, cemented prosthesis during the initial surgery. This construct is designed to maintain joint stability, allow full weight-bearing, and preserve range of motion, thereby minimizing the morbidity associated with prolonged spacer use [[15], [16], [17], [18]]. The terminology for this procedure is inconsistent in the literature, with a 1.5-stage arthroplasty also referred to as a “Kiwi procedure” or permanent articulating spacer. It mainly differs from a single-stage revision performed for periprosthetic infection in the cementing technique, which facilitates easier removal if revision or progression to a second stage becomes necessary.

Importantly, this approach preserves the option for future reimplantation of a more definitive prosthesis—aimed at optimizing joint function and mobility with a well-fixed implant, including restoration of range of motion, strength, and the ability to perform daily, occupational, and recreational activities—while allowing selected patients, particularly those who are low-demand or medically frail, to safely avoid a second-stage procedure [14,19].

Emerging evidence suggests that the 1.5-stage technique achieves infection eradication rates comparable to those of the traditional 2-stage approach in the management of PJI, while offering the added benefits of fewer complications, shorter hospital stays, and improved functional outcomes [[20], [21], [22], [23], [24]].

While this approach is well-established in PJI management, its role in NJSA remains underexplored. The aim of this study was to explore the outcomes of 1.5-stage arthroplasties in hips and knees for NJSA in selected patients with extensive joint destruction, difficulty with source control or severe pre-existing degenerative joint disease.

## Material and methods

### Study design and patient population

This retrospective cohort study included a consecutive series of patients who presented to our orthopaedic department at a tertiary public hospital with NJSA in the context of degenerative hip or knee joint disease between February 2015 and January 2024. All adult patients who underwent a 1.5-stage arthroplasty were included. We retrospectively collected data from the departmental orthopaedic infection database and electronic medical records, including patient charts, operative reports, discharge summaries, clinic letters, and patient-reported outcome measures (PROMs). This study received institutional review board approval at our institution prior to commencement (GEKO number: 47608).

### Outcome measures and data collection

The primary outcome was the revision rate at 12 months follow-up, defined as any additional surgical procedure involving component exchange after implantation of the 1.5-stage arthroplasty.

Secondary outcomes included the incidence and types of postoperative surgical complications, retention of 1.5 arthroplasty, length of hospital stay, 30-day emergency department visits and hospital readmissions, and mortality. Furthermore, PROMs, including the Oxford Knee Score, Oxford Hip Score, and the Forgotten Joint Score (FJS), were collected at the 1-year follow-up, when possible. Surgical complications were defined as any event necessitating an unplanned reoperation. The 12-month endpoint was defined as the period up to 1 year following the 1.5-stage arthroplasty operation.

Baseline demographic and clinical variables included age at surgery, sex, American Society of Anesthesiologists classification, body mass index, smoking status, intravenous drug use, Charlson Comorbidity Index [25], Clinical Frailty Scale [26], mode of acquisition of infection, microbiological and biochemical characteristics of the initial joint aspirate, and the type and duration of antibiotic treatment.

### Patient baseline data

We identified 45 patients treated for NJSA associated with degenerative hip (n = 27) or knee (n = 18) joint disease who underwent a 1.5-stage arthroplasty. Table 1 provides an overview of patient demographics and clinical characteristics. The mean age was similar in both the hip and knee cohorts (69.0 vs 73.5 years). The hip cohort was characterized by greater frailty, a higher comorbidity burden, and a higher proportion of women.

**Table 1:** Overview of patient demographics and clinical characteristics.

Characteristic	Total n = 45	Hip cohort n = 27	Knee cohort n = 18	*P* value
Age, in years, median (IQR)	72.0 (60-80)	69.0 (59.5-79.5)	73.5 (62.5-79.0)	.799^a^
Male sex, n (%)	30 (66.7%)	14 (51.9%)	16 (88.9%)	.012^b^
BMI, median (IQR)	28.5 (25.3-32.4)	29.6 (24.7-36.0)	28.3 (25-7-30.1)	.537^a^
ASA score, median (IQR)	3.0 (2.0-3.0)	3.0 (2.25-3.0)	2.0 (2.0-3.0)	.061^a^
Current smoker, n (%)	6 (13.3%)	5 (18.5%)	1 (5.6%)	.353^b^
Active IVDU, n, (%)	1 (0.2%)	1 (3.7%)	0 (0%)	1.0^b^
Charlson Comorbidity Index, median (IQR)	3.0 (2-5)	4.0 (2.0-5.0)	3.0 (2.0-4.75)	.664^a^
Clinical Frailty Scale, median (IQR)	3.0 (2-4)	4.0 (3.0-5.0)	2.5 (2.0-3.0)	.001^a^
Indication				
Difficulty with source control leading to extensive joint destruction	19 (42.2%)	5 (18.5%)	14 (77.8%)	.005^b^
Severe pre-existing OA	26 (57.8%)	22 (81.5%)	4 (22.2%)	.005^b^
Mode of acquisition				.661^b^
Hematogenous, n (%)	13 (28.9%)	9 (33.3%)	4 (22.2%)	
Direct inoculation, n (%) (through CSI)	20 (44.4%) (13)	10 (37.0%) (7)	10 (55.6%) (6)	
Unknown, n (%)	12 (26.7%)	8 (29.6%)	4 (22.2%)	
Concomitant crystal arthropathy, n (%)	11 (24.4%)	6 (22.2%)	5 (27.8%)	.732^b^
Diagnosis to arthroplasty, in days, median (IQR)	6 (2-12.8)	4 (2-7)	9 (6-17)	.075^a^
Debridement prior to arthroplasty				
Total, n (%)	19 (42.2%)	5 (18.5%)	14 (77.8%)	.005^b^
Open, n (%)	8 (17.8%)	5 (18.5%)	3 (16.7%)	
Arthroscopic, n (%)	11 (24.4%)	0 (0%)	11 (61.1%)	
Antibiotics duration				
IV antibiotics duration; in weeks, median (IQR)	6 (4-6)	6 (4-6)	6 (4-6)	.639^a^
Oral antibiotics duration; in weeks, median (IQR)	6 (4-12)	6 (4-12)	6 (5.5-10.5)	.865^a^
Chronic suppressive antibiotic therapy, n (%)	7 (15.6%)	5 (18.5%)	2 (11.1%)	.454^b^

ASA, American Society of Anesthesiologists; BMI, body mass index; CSI, corticosteroid injection; IQR, interquartile range; IV, intravenous; IVDU, intravenous drug use; OA, osteoarthritis.

a Mann-Whitney U test.

b Fisher's exact test.

Causative pathogens (Table 2) and the occurrence of concomitant crystal arthropathy were comparable between cohorts. In total, 20 (44.4%) acquired their infection via direct inoculation, including 13 following corticosteroid injections and 7 as a result of previous surgery or direct traumatic inoculation. Hematogenous spread was the most frequent mode of acquisition in the hip cohort, while direct inoculation predominated in the knee cohort. In both cohorts, hematogenous infections most commonly arose secondary to urosepsis, infected lower limb ulcers, or abscesses involving other musculoskeletal structures.

**Table 2:** Overview of identified pathogens.

Characteristic	Total n = 45	Hip cohort n = 27	Knee cohort n = 18	*P* value
Identified pathogens				.985^a^
Monomicrobial, n (%)	28 (62.2%)	17 (63.0%)	11 (61.1%)	
Polymicrobial, n (%)	7 (15.6%)	4 (14.8%)	3 (16.7%)	
Culture negative, n (%)	10 (22.2%)	6 (22.2%)	4 (22.2%)	
*Staphylococcus aureus*, n	17	8	9	
*MSSA*	16	7	9	
*MRSA*	1	1	0	
*Staphylococcus epidermidis*, n	8	5	3	
*Staphylococcus capitis*, n	3	1	2	
*Streptococcus agalactiae,* n	2	2	0	
*Staphylococcus caprae,* n	2	1	1	
*Streptococcus dysgalactiae*, n	1	1	0	
*Streptococcus mitis,* n	1	1	0	
*Serratia marcescens* n	1	0	1	
*Escherichia coli*, n	1	0	1	
*Enterococcus faecalis,* n	1	1	0	
*Salmonella spp,* n	1	1	0	
*Moraxella catarrhalis,* n	1	0	1	
*Citrobacter koseri,* n	1	1	0	
*Parvimonas micra,* n	1	1	0	
*Candida spp.,* n	1	1	0	
*Scedosporium ssp.,* n	1	0	1	

MSSA, methicillin-sensitive *Staphylococcus aureus*; MRSA, methicillin-resistant *Staphylococcus aureus*; ssp, subspecies.

a Fisher's exact test.

Less than half of the patients (42.2%) had a surgical debridement prior to the decision to proceed to arthroplasty.

An overview of identified pathogens is presented in Table 2.

### Surgical technique

Native joint aspiration was underdone upon initial presentation to confirm infection in all patients. Arthroscopic or open debridement was performed prior to arthroplasty in cases with an acute presentation and no or little radiographic evidence of osteoarthritis. In cases of failed source control with extensive joint destruction, a 1.5-stage arthroplasty was performed, with the decision driven by the extent of structural damage—such as severe osteoarthritis, instability, subchondral fracture, collapse, or femoral head dissolution—where joint preservation was considered unlikely.

**Figure 1:**
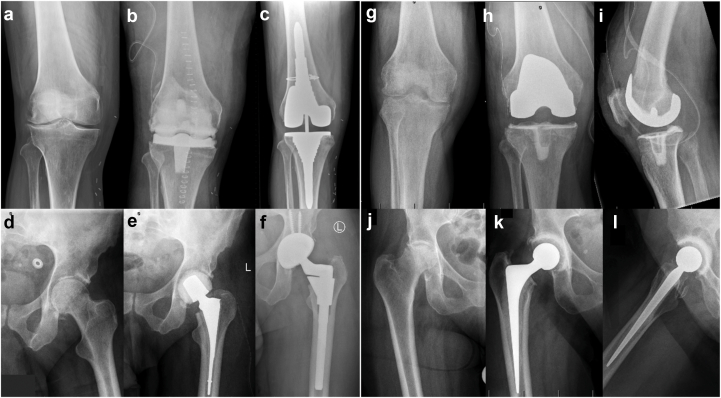
Serial radiographs demonstrating the 2 different treatment approaches for knee and hip NJSA. Two-stage arthroplasty (left): Preoperative images (a, d), postoperative images following spacer implantation (b, e), and final radiographs after second-stage implantation (c, f). 1.5-stage arthroplasty (right): Preoperative images (g, j), postoperative anteroposterior views (h, k), and lateral views (i, l) showing the definitive implant.

Patients with severe pre-existing osteoarthritis proceeded directly to 1.5-stage arthroplasty whenever feasible. All patients received antibiotic treatment tailored to the identified pathogen, following an interdisciplinary approach in collaboration with the infectious disease department. The 1.5-stage arthroplasty involved thorough debridement of infected and necrotic tissue, including synovectomy. Tissue samples were obtained intraoperatively for microbiological analysis. Rather than using a temporary spacer, a cemented prosthesis with antibiotic-loaded cement was implanted during the index procedure [13,14]. The fixation depends on molding the cement mantle to fit the general contours of the bone, rather than achieving the deep microscopic interlock with cancellous bone seen in standard cemented arthroplasty. This technique allows the cement to adhere to the surface without extensive penetration, making implant removal more straightforward if a second-stage revision is required [14]. In hips, an appropriately sized acetabular polyethylene liner is embedded into the cement during its doughy phase, and the metal femoral component is similarly surrounded by doughy cement before insertion. A metal femoral head was used in all cases. In knees, a rotating platform polyethylene liner and a cruciate-retaining metal femoral component were used with the same cementing technique. These constructs provided immediate joint stability and function, with the option of later conversion to a second-stage revision if required for mechanical or functional reasons (Fig. 1, right). Patients treated with a 1.5-stage arthroplasty were followed at regular intervals with clinical assessments and radiographs to evaluate for loosening or subsidence. Those with worsening symptoms, declining function in daily, occupational, or recreational activities, or progressive radiographic loosening were counseled regarding a potential second-stage procedure. Decisions were made on an individual basis using a shared decision-making approach, incorporating clinical findings, radiographic progression, and patient-reported outcomes.

### Antibiotic therapy

All patients received an initial course of intravenous antibiotics followed by oral therapy. The choice and duration of antibiotics were determined by culture and sensitivity results, host factors, and clinical response. Rifampicin was prescribed in 1 patient (2.2%). Antibiotic suppressive therapy was recommended by the attending infectious diseases clinician on a case-by-case basis in situations where durable, definitive cure was unlikely. Infection eradication was defined by normalization of inflammatory markers (C-reactive protein <10 mg/L), absence of wound complications, unexplained fever or bacteremia, and no clinical evidence of purulence around the prosthesis [27]. Inflammatory markers were monitored regularly in the outpatient setting and by the general practitioner. Suspected reinfection was confirmed microbiologically: growth of the original index organism (same genus, species, and antibiogram, if available) in ≥1 culture was considered significant, whereas new organisms required ≥2 positive specimens.

### Data analyses

Descriptive analyses summarized patient characteristics. Continuous variables were reported as medians with interquartile ranges and compared using the Mann–Whitney U test. Baseline characteristics were summarized with statistical comparison between hips and knees. To identify potential factors associated with the need for revision, univariate analyses were performed. Binary outcomes were expressed as relative frequencies and compared using Fisher's exact test. Ordinal and continuous outcomes were analyzed with the Mann–Whitney U test. Statistical analyses were performed using Jamovi v2.3.3.0 (Sydney, New South Wales, Australia). All *P* values <0.05 were considered significant.

## Results

At 1 year, the revision rate was 26.7%, with only 2 revisions performed for surgical complications. One patient experienced reinfection and was revised with conversion to a cement spacer, and 1 patient sustained a hip dislocation requiring revision to a second stage. Overall, complications were infrequent; aside from the revision cases, 1 patient underwent superficial soft tissue debridement and closure for wound dehiscence with retention of the 1.5-stage arthroplasty. All surgical complications occurred within the hip cohort.

The mean length of stay and hospital re-presentation rates did not differ between cohorts. At 1 year, 75.6% of 1.5-stage arthroplasties had retained their destination implant. Mortality rates were comparable across cohorts. Table 3 summarizes the differences in primary and secondary outcomes between the hip and knee cohorts.

**Table 3:** Results of primary and secondary outcome measures.

Outcome	1.5-stage cohort n = 45	Hip cohort n = 27	Knee cohort n = 18	*P* value
Revision surgery at 1 year, n (%)	12 (26.7%)	8 (29.6%)	4 (22.2%)	.735^a^
Surgical complications total, n (%)	3 (6.67%)	3 (16.7%)	0 (0%)	.143^a^
Reinfection, n (%)	1 (2.2%)	1 (5.6%)	0 (0%)	
Instability, n (%)	1 (2.2%)	1 (5.6%)	0 (0%)	
Wound dehiscence, n (%)	1 (2.2%)	1 (5.6%)	0 (0%)	
Progression to second stage, n (%)	11 (24.4%)	7 (25.9%)	4 (22.2%)	1.0^a^
Length of stay; in days, median (IQR)	13.5 (8-16)	14 (8.5-16)	13 (7.5-17)	.654^b^
Hospital re-presentation				
30-d ED presentation, n (%)	5 (11.1%)	4 (14.8%)	1 (5.6%)	.333^a^
30-d readmission, n (%)	3 (6.7%)	3 (16.7%)	0 (0%)	.143^a^
Mortality, n (%)	2 (4.4%)	1 (5.6%)	1 (3.7%)	.768^a^

ED, emergency department; IQR, interquartile range.

a Fisher's exact test.

b Mann-Whitney U test.

Of the 45 patients who underwent 1.5-stage arthroplasty, 24.4% (n = 11) progressed to a formal second stage within 1 year. This group included one patient who sustained a hip dislocation requiring revision. Other indications for conversion included planned second-stage revision due to high functional demand (n = 6), knee stiffness (n = 2), and leg length discrepancies in hip cases (n = 2). Postoperative knee stiffness was defined as a total range of motion less than 90° or a flexion contracture greater than 10°, resulting in functional limitation. In all patients who underwent a formal second-stage procedure, intraoperative cultures obtained to rule out persistent infection were negative.

PROMs at 1-year follow-up were available for 19 of 45 patients (42.2%), with a mean Oxford score of 34.7 and a mean FJS of 46.9. Table 4 summarizes PROMs for the hip and knee cohorts.

**Table 4:** Patient-reported outcome measures at 1-year follow-up.

PROMS	Total n = 45	Hip cohort n = 27	Knee cohort n = 18
Patients with PROMS, n	19 (42.2%)	12 (44.4%)	7 (38.9%)
FJS, median (IQR)	37.5 (20.8-75.0)	47.9 (20.8-80.7)	37.5 (24.0-39.6)
Oxford score, mean (IQR)	40.0 (29.0-43.0)	40.0 (28.3-44.3)	30.0 (29.0-37.0)

FJS, Forgotten Joint Score; IQR, interquartile range; PROMS, patient reported outcome measures.

Univariable logistic regression revealed no significant associations between baseline characteristics and revision risk (Table 5).

**Table 5:** Univariable association of risk factors with revision of 1.5 arthroplasty.

Characteristic	Odds ratios	95% CI	*P* value
Age	0.993	0.948-1.04	.748
Male sex	3.250	0.611-17.283	.167
BMI	1.005	0.921-1.10	.912
ASA score	0.458	0.157-1.34	.153
Current smoker	1.964	0.206-18.78	.558
Charlson Comorbidity Index	1.063	0.735-1.54	.746
Clinical Frailty Scale	0.564	0.296-1.07	.082
Concomitant crystal arthropathy	1.04	0.226-4.81	.958
Debridement prior to arthroplasty	0.381	0.031-4.55	.446
Culture negative	1.450	0.23-9.16	.693

ASA, American Society of Anesthesiologists; BMI, body mass index.

## Discussion

This study demonstrates that 1.5-stage arthroplasty offers a viable and effective alternative to traditional 2-stage arthroplasty for managing NJSA in the setting of degenerative hip and knee disease. Our findings indicate that the 1.5-stage approach was associated with low reinfection rates, few surgical complications, and short hospital stays. These results suggest that 1.5-stage arthroplasty may provide comparable infection control while minimizing the morbidity and healthcare burden associated with multiple procedures.

Our study demonstrated a total reinfection rate of 2.2% at 12 months in patients who received a 1.5-stage arthroplasty. Importantly, these favorable results were achieved despite inclusion of patients with culture-negative and polymicrobial infections, traditionally considered more challenging to manage [28].

Historically, 2-stage arthroplasty has been regarded as the gold standard for managing NJSA in patients with severe pre-existing osteoarthritis, with current literature reporting infection-free survival rates between 84% and 100% for hips and knees treated with this approach [7,[29], [30], [31], [32], [33], [34], [35], [36], [37], [38]]. Xu et al. evaluated 2-stage arthroplasty success and risk factors for failure in 74 hip and knee NJSA cases, with a mean follow-up of 4.7 years [38]. Two-year survivorship was 90.7% for hips and 89.5% for knees. Older age, elevated preoperative C-reactive protein, and resistant organisms were key risk factors for failure. Kunze et al. reported an infection cure rate of 95.2%, accompanied by a high complication rate of 33% in their cohort of 42 patients undergoing 2-stage arthroplasty for NJSA [32]. Similar findings were reported by Chen et al. [7] and Russo et al. [30], with both studies demonstrating infection eradication rates greater than 90%, but overall complication rates ranging from 27.7% to 36%.

In our series, the 1.5-stage cohort demonstrated a low surgical complication rate of 4%. In contrast, reported complication rates for 2-stage procedures reach up to 36%, largely driven by mechanical issues such as spacer dislocation, spacer fracture, bone loss associated with prolonged spacer use, and periprosthetic fracture. These complications often require additional surgical interventions and may delay recovery [7,30,[36], [37], [38], [39]].

Another advantage of the 1.5-stage approach observed in our study was a short hospital length of stay (median 13.5 days). This likely reflects the avoidance of prolonged interim periods with a temporary spacer and the reduced need for additional surgical interventions. Reducing surgical episodes and hospitalization has significant economic benefits for both health systems and patients. The recently published INFection ORthopaedic Management (INFORM) trial in hip PJI similarly highlighted the cost savings and reduced perioperative morbidity associated with single-stage strategies [40].

In our study, only 24.4% of patients in the 1.5-stage arthroplasty cohort proceeded to a second stage, primarily for functional optimization. This was defined as optimizing joint function and mobility with a well-fixed prosthesis, with the goal of restoration of range of motion, strength, and the ability to perform daily, occupational, and recreational activities. The second-stage procedures in these patients can therefore be seen as planned, expected procedures rather than failures or true complications. The remaining 33 patients (73.3%) retained their initial implant, either because they were satisfied with the functional outcome or were unfit for further surgery. Similar to PJI management, this temporal flexibility is a key strength of the 1.5-stage strategy, particularly for medically frail patients or those with low functional demand who may not tolerate multiple procedures.

Although PROMs were available for less than half of all included patients, the 1.5-stage cohort demonstrated satisfactory Oxford and FJSs at 1 year. Previous studies have found no difference in PROMs between 1.5- and 2-stage arthroplasty for chronic periprosthetic hip infections [41]. These results should be interpreted with caution, given the small sample size and the heterogeneity in treatment pathways, as some patients were expected to undergo a second-stage procedure while others were not. This variability precluded adjustment for potential confounders such as age, comorbidities, and preoperative functional status.

In our cohort, univariable analysis did not reveal any risk factors significantly associated with revision, suggesting that early revision was not strongly predicted by any single factor. Specifically, higher American Society of Anesthesiologists scores (OR = 0.458, *P* = .153) and Clinical Frailty Scale (OR = 0.564, *P* = .082) were not associated with revision, confirming that sicker and frailer patients achieve favorable outcomes.

One-stage arthroplasty may also represent a potential treatment alternative for patients with NJSA and associated degenerative joint disease, offering comparable infection control with lower perioperative morbidity and cost, but its use is typically reserved for carefully selected patients [6,[42], [43], [44]]. In addition, failure of a 1-stage arthroplasty poses a particular challenge, as removal of a well-fixed implant often necessitates extensive soft tissue and bone resection.

The 1.5-stage approach offers a balance between the benefits of 1-stage and 2-stage arthroplasty. By implanting an antibiotic-loaded, cemented prosthesis during the initial surgery, it provides immediate joint stability and allows early mobilization and weight-bearing [12,45]. Unlike temporary spacers, these components can serve as a definitive solution or as a platform for later conversion to a second-stage revision if required. Moreover, implant removal is generally easier and more bone-preserving compared to 1-stage arthroplasty.

The authors consider the 1.5-stage approach appropriate for patients with native septic joint arthritis of the hip and knee who have adequate bone stock for fixation and a satisfactory soft tissue envelope. In the present study, this strategy provided reliable infection control with low reinfection rates. For patients with higher functional demands, a planned second stage with implantation of a definitive prosthesis remains possible, allowing better interim mobility and function. Importantly, 3-quarters of patients in our cohort retained their 1.5-stage arthroplasty without requiring additional surgery. By contrast, a conventional 2-stage approach would not provide this advantage, as the limited functional capacity of a cement spacer often results in restricted mobility between stages.

### Limitations

This study has several limitations. Follow-up was restricted to 12 months; while this period is sufficient to capture most early reinfections, longer-term outcomes regarding prosthesis survival and late infection recurrence require further study. Preinfection functional status was not evaluated, but could impact both outcomes and satisfaction, with highly active patients potentially less satisfied with the 1.5-stage approach. The small cohort size may have limited our ability to identify statistically significant risk factors for revision. In addition, the collection of PROMs was incomplete, with data available for only 50% of patients, and should therefore be interpreted with caution. The small numbers in the hip and knee subgroup analyses further limit the reliability and generalizability of these findings.

## Conclusions

Our findings demonstrate that 1.5-stage arthroplasty represents a safe and effective treatment option for NJSA in patients with degenerative hip or knee disease, offering low reinfection rates, few complications, short hospital stays, and the potential to avoid additional surgery. This approach may be particularly valuable for medically frail or low-demand patients, or in cases where multiple operations pose significant risk. Future prospective studies with larger cohorts and longer follow-up and systematically collected PROMs are needed to validate these results and define the optimal indications and long-term performance of the 1.5-stage strategy in NJSA.

## CRediT authorship contribution statement

**Matthias Wittauer:** Writing – review & editing, Writing – original draft, Validation, Resources, Methodology, Investigation, Formal analysis, Data curation. **Conor Farrell:** Writing – original draft, Investigation, Formal analysis, Data curation. **Nikhil Sabharwal:** Investigation, Formal analysis, Data curation. **Laurens Manning:** Writing – review & editing, Validation, Supervision, Methodology, Conceptualization. **Benjamin Clark:** Writing – review & editing, Methodology, Investigation. **Piers J. Yates:** Writing – review & editing, Validation, Supervision, Conceptualization. **Christopher W. Jones:** Writing – review & editing, Supervision, Project administration, Methodology, Investigation, Conceptualization.

## Conflict of interest

C.W. Jones receives royalties from DePuy Synthes; is on the speakers bureau/paid presentations for Medacta and MatOrtho; is a paid consultant for DePuy Synthes and NavBit; is an unpaid consultant for MatOrtho; has stock or stock options in NavBit Pty Ltd; and receives research support from DePuy Synthes and Zimmer Biomet. P.J. Yates receives royalties from Corin and MatOrtho; is on the speakers bureau/paid presentations for DePuy and MatOrtho; is a paid consultant for DePuy and MatOrtho; has stock or stock options in Eventis; receives research support from DePuy and Corin; is a member of the medical/orthopaedic publications editorial/governing board at Hip international; and is a board member/committee appointments for AOA.

For full disclosure statements refer to https://doi.org/10.1016/j.artd.2026.102038.

## Appendix A Supplementary data

Conflict of Interest Statement for Clark

Conflict of Interest Statement for Farrell

Conflict of Interest Statement for Jones

Conflict of Interest Statement for Sabharwal

Conflict of Interest Statement for Wittauer

Conflict of Interest Statement for Yates

Conflict of Interest Statement for Manning
