# Supplementary material for: Clinical Outcomes of 1.5-Stage Arthroplasty for Native Joint Septic Arthritis of the Hip and Knee: A Retrospective Cohort Study
Source: Arthroplast Today. 2026 May 20;39:102038. doi: 10.1016/j.artd.2026.102038 (PMC13213679; doi:10.1016/j.artd.2026.102038)
Supplement: Conflict of Interest Statement for Yates [file mmc6.pdf]

INDIVIDUAL CONFLICT OF INTEREST STATEMENT

American Association of Hip and Knee Surgeons

(Adopted from the American Academy of Orthopaedic Surgeons disclosure statement)

The following form **must be filled out completely and submitted by each author (example, 6 authors, 6 forms). All items require a response. If there is no relevant disclosure for a given item, enter "*None.*"**

Manuscript Title: Clinical Outcomes of 1.5-Stage Versus 2-Stage Arthroplasty for Native Joint Septic Arthritis: A Retrospective Cohort Study

1. Royalties from a company or supplier (The following conflicts were disclosed)
Corin, matortho

2. Speakers bureau/paid presentations for a company or supplier (The following conflicts were disclosed)
Depuy, matortho

3A. Paid employee for a company or supplier (The following conflicts were disclosed)
None

3B. Paid consultant for a company or supplier (The following conflicts were disclosed)
Depuy, matortho

3C. Unpaid consultants for a company or supplier (The following conflicts were disclosed)
None

4. Stock or stock options in a company or supplier (The following conflicts were disclosed)
Eventis

5. Research support from a company or supplier as a Principal Investigator (The following conflicts were disclosed)
Depuy, corin

6. Other financial or material support from a company or supplier (The following conflicts were disclosed)
None

7. Royalties, financial or material support from publishers (The following conflicts were disclosed)
None

8. Medical/Orthopaedic publications editorial/governing board (The following conflicts were disclosed)
Hip international

9. Board member/committee appointments for a society (The following conflicts were disclosed)
AOA

Each author must sign AND print or type his/her name, date and submit a separate form

In addition, one BLINDED Conflict of Interest form (no author names used) should be submitted per manuscript with all author disclosures.

Piers Yates	[signature]	11/14/2025
Author Name (Print or Type)	Author Signature	Date
